# The Role of Oxidative Stress and the Effects of Antioxidants on the Incidence of Infectious Complications of Chronic Lymphocytic Leukemia

**DOI:** 10.1155/2014/158135

**Published:** 2014-10-14

**Authors:** Amelia Maria Gaman, Ana-Maria Buga, Mihnea-Alexandru Gaman, Aurel Popa-Wagner

**Affiliations:** ^1^Research Center of Experimental and Clinical Medicine, University of Medicine and Pharmacy of Craiova, Romania; ^2^Filantropia City Hospital of Craiova, Romania; ^3^Carol Davila University of Medicine and Pharmacy, Bucharest, Romania; ^4^Department of Psychiatry, University of Medicine Rostock, Gehlsheimerstrasse 20, 18147 Rostock, Germany

## Abstract

Chronic lymphocytic leukemia (CLL) is characterized by a predominant humoral immune deficiency predisposing the patients to infections. Oxidative stress leads to an increased immunoglobulin k light chain production in B cells and contributes to the antibodies' deficiency and hypogammaglobulinemia. *Aim of the Study*. To evaluate the global oxidative status in patients with CLL and to determine whether the administration of antioxidants decreases complications due to infections. *Patients and Method*. We studied 84 patients with CLL stratified by Binet staging. Free oxygen radicals and antioxidant status were determined by the FORT and FORD test, respectively, at diagnosis and in the presence of infections. The patients were distributed in two groups: group A, treated only with antileukemic treatment, and group B, treated with antileukemic treatment and antioxidants. *Results*. By FORD and FORT assay, all patients had at diagnosis a low antioxidant capacity, and high levels of hydroperoxides. Infectious complications were more frequent in group A (B/C stages of disease) than in group B. Administrations of antioxidants stimulated the immune response and decreased infectious complications in CLL. *Conclusions*. Administrations of antioxidants and a healthy life style may improve the quality of life of patients with CLL and reduce the risk of infectious complications.

## 1. Introduction

The reactive oxygen species (ROS), superoxide radical anion, hydrogen peroxide, or hydroxyl radical, are produced continuously during basal cellular metabolism, frequently in the mitochondria or in other cellular compartments, due to the action of oxidases. ROS also appear in antioxidant deficiency, mitochondrial dysfunction, inflammation, phagocytosis (myeloperoxidase activity), exogenous stress (exogenous oxidants, redox cycling agents, UV irradiation, chemicals, endotoxins, and hyperoxia) [1–4]. ROS alter biological macromolecules (DNA, carbohydrates, proteins, and lipids).

The antioxidant systems act as ROS scavengers. For example, superoxide dismutase and catalase convert hydrogen peroxide to superoxide and then to molecular oxygen. Various peroxide compounds, including hydrogen peroxide, can be inactivated by the systems composed of glutathione peroxidase, glutathione reductase, and glucose-6-phosphate dehydrogenase. Vitamins C and E also act as radical scavengers [5, 6].

Immune cell functions are linked to ROS production and antioxidant defense. Therefore, antioxidant deficiency can be one cause of immune function suppression, affecting both innate T-cell-mediated immune response and adaptive antibody response [7].

Disturbances of oxidative stress metabolism are a common feature of transformed tumour cells. Most cancer cells are active in the metabolic production of ROS [8]. Moreover, ROS-related lesions that do not cause cell death can stimulate the development of cancer. Therefore, ROS is an important class of carcinogens involved in mutagenesis through oxidative DNA damage, the expansion of tumour clones, and the acquisition of malignant properties [9]. In particular, malignant lymphocytes from CLL patients have been demonstrated to produce abundantly superoxide anions [10].

Oxidative stress induces NFkB (nuclear factor inducing immunoglobulin k light chain production in B cells), increases immunoglobulin k light chain production in B cells, and contributes to antibodies' deficiency and to hypogammaglobulinemia [9]. A healthy diet, rich in natural antioxidants (vitamin C, vitamin E, selenium, zinc, and copper), decreases the free radicals level in the human body [10–12].

On the basis of all this data,we evaluated the global oxidative status in patients with chronic lymphocytic leukemia and asked whether the administration of antioxidants decreases infectious complications in these patients.

## 2. Patients and Method

84 patients with CLL hospitalized in the Clinic of Hematology were recruited for the study. This study was conducted in accordance with the updated Declaration of Helsinki and was approved by the Ethics Committee. Informed consent was obtained from all patients prior to study enrollment, according to the ethical code.

The patients were stratified by age, sex, urban/rural environment, and status (smokers or nonsmokers). The biological parameters determined were hemoglobin value, reticulocytes, leukocyte count and leukocyte formula, platelet count, peripheral blood smear, bone marrow smear, erythrocytes sedimentation rate, acute phase proteins (fibrinogen and reactive C protein), electrophoresis, immunoelectrophoresis, uric acid level, Coombs test in some cases, hepatic and renal tests, glycemia, and lipid profile. The diagnosis of CLL was established clinically, biologically, and morphologically and by immunophenotyping. The classification was done according to the Binet staging. The comorbidities evaluated were arterial hypertension, respiratory diseases, diabetes mellitus, lipid disorders, severe liver diseases, kidney failure, alcoholism, and a second neoplasm. The antileukemic treatment consisted of Chlorambucil + Prednisone, CVP (Cyclophosphamide + Vincristine + Prednisone) regimen, Fludarabine, RFC (Rituximab + Fludarabine + Cyclophosphamide) regimen, or alemtuzumab (anti-CD52 monoclonal antibody). The diagnosis of the infectious complications was established on the basis of fever, systemic, or local infectious signs, X-ray, and bacteriological or virusological exams. The global oxidative status was evaluated on all patients at the diagnosis of CLL and in the presence of infections using a CR3000 analyzer (Callegari SpA). Both free oxygen radicals and antioxidant status were evaluated by FORT and FORD tests from a single drop of capillary blood [12–15]. The FORT test is a colorimetric test based on the ability of transition metals (iron) to catalyze the breakdown of hydroperoxides into derivative radicals according to the Fenton reaction. Once formed in cells, the organic hydroperoxides (ROOH) maintain their chemical reactivity and oxidative capacity to generate proportional amounts of alkoxyl and peroxyl radicals which are preferentially trapped by a suitably buffered chromogen and at 37°C develop a coloured cation which is detectable photometrically. The intensity of the colour correlates directly with the quantity of radical compounds and can be related to the oxidative status of the sample. The normal ranges of FORT are up to 310 Fort units or 2.3 mmol/L H_2_O_2_. The FORD test is a colorimetric test based on the ability of antioxidants present in plasma to reduce a preformed radical cation. A stable and coloured cation is formed in the presence of an acidic buffer, at a pH of 5.2, and a suitable oxidant, FeCl_3_, and is detectable photometrically at 505 nm. Antioxidant compounds present in the sample reduce the radical cation of the chromogen, thus quenching the colour and producing a decoloration of the solution which is proportional to their quantity in the sample. The normal ranges of FORD are 1.07–1.53 mmol/L. The patients were divided into two groups: group A, 44 patients with CLL who received only antileukemic treatment, and group B, 40 patients who received antileukemic treatment and antioxidants (vitamin C). Statistical analysis was performed and a *P* value ≤0.05 was considered significant.

## 3. Results

The median age of the patients with CLL was of 65 years; the sex ratio, M/F = 1.62, revealed a male predominance. Patient stratification by Binet staging showed the following: CLL-stage A, 23 patients, CLL-stage B, 30 patients, and CLL-stage C, 31 patients. Immunophenotyping revealed B phenotype in 80 cases and T phenotype in 4 cases. Characteristics of patients with CLL for group A (44 patients) and group B (40 patients) are presented in Table 1.

The age of the patients from group A (predominantly males) varied between 47 and 85 years; 42 patients had B cell phenotype (B-CLL) and 2 patients T-cell phenotype (T-CLL). 13 patients were in stage A of the disease, 15 in stage B, and 16 in stage C of disease. All patients had at diagnosis low FORD values (between 0.48 and 0.96 mmol/L) and high FORT values (between 2.7 and 3.8 mmol/L). Uric acid values were normal in 28 cases and high in 16 cases (Table 2). Nineteen patients were smokers and twenty-five nonsmokers. Three patients had arterial hypertension, three had diabetes mellitus, five had respiratory diseases, two had liver diseases, one had kidney failure, and four had secondary cancers; two basocellular epithelioma, one colorectal cancer and one bladder cancer. The patients were treated with Chlorambucil + Prednisone in 10 cases, CVP regimen in 10 cases, Fludarabine in 5 cases, RFC in 4 cases, and alemtuzumab in 2 cases (Table 3). Twenty-two patients had infectious complications of whom, twelve had viral pneumonia, four had bacterial pneumonia, three had urinary infections, two with cutaneous staphylococcus and one with pleurisy. Hypogammaglobulinemia was present in all cases and granulocytopenia in 60% of the cases, explaining the high frequency of infections (Table 4).

The age of the patients from group B varied between 51 and 84 years old with a male prevalence; 38 patients had B-CLL and 2 patients had T-CLL. Ten patients were in the stage A of disease, fifteen patients in stage B, and fifteen in stage C of disease (Table 1). All patients had at diagnosis low FORD values (between 0.42 and 0.89 mmol/L) and high FORT values (between 2.5 and 3.2 mmol/L). Acid uric values were normal in 34 cases and high in 6 cases (Table 2). Sixteen patients were smokers and twenty-four nonsmokers. Three of them had arterial hypertension, two had respiratory diseases, one had diabetes mellitus, two had dyslipidemic syndrome, and two had secondary cancers of whom one with basocellular epithelioma and one with prostatic cancer one basocellular epithelioma and one prostatic cancer. The patients were treated with Chlorambucil + Prednisone in ten cases, CVP regimen in nine cases, fludarabine in five cases, RFC in four cases, and alemtuzumab in two cases. They received 1000 mg of vitamin C daily, for five days monthly, during the period of the therapeutic regimens. They were nonsmokers, did not drink alcohol, and had a healthy diet with fruit, vegetables, nuts, and seeds (Table 3). Five patients had infectious complications, viral pneumonia in two cases, bacterial pneumonia in one case, and urinary infection in two cases. Hypogammaglobulinemia was present in 75% of cases (Table 4).

## 4. Discussion

Malignant lymphocytes from CLL patients have been demonstrated to produce abundantly superoxide anions [10]. In this study, we have shown that administrations of antioxidants and a healthy life style stimulated the immune response and the tolerance to chemotherapy and decreased infectious complications in CLL.

Human cells are exposed to a large variety of ROS from both exogenous (radiation exposure, air pollutants, industrial contaminants, drugs, cigarette smoke, alcohol, pesticides, herbicides, and food) and endogenous sources (energy metabolism). On the other hand, the antioxidant capacity of the human body decreases with age [16].

Oxidative stress is involved in a variety of human diseases and disorders, including apoptosis and carcinogenesis. The data about oxidative stress in CLL is controversial: in the early stages of CLL, an imbalance between the mechanisms that generate ROS and the antioxidant defense mechanisms in favour of the latter may occur; in the late stages of CLL, malignant cells are generally more active in the production of ROS than normal cells mainly because of the mitochondrial defects caused by chemotherapy [17–19].

Patients from both groups had at diagnosis low FORD values (antioxidant capacity testing) and high FORT values (free oxygen radicals testing). Low FORD values at diagnosis indicate a decreased antioxidant capacity in patients over 60 years old with CLL that could be in part due to the natural decrease in the antioxidant capacity with increasing age. High FORT values highlight a high level of ROS, especially in the advanced stages of disease (B and C), when malignant lymphocytes are more active in the production of ROS, a process exacerbated by aggressive chemotherapy. The association of comorbidities (diabetes mellitus, severe hepatic diseases, and chronic alcoholism) increased the level of ROS as well. Initially, the high FORT values in patients from the B group decreased after the administration of exogenous antioxidants which, presumably, have neutralized the ROS.

Some of the patients had high levels of uric acid (16 cases in group A versus 6 cases in group B). Under physiological conditions, uric acid has a powerful antioxidant activity, being able to directly scavenge free radicals but becomes prooxidant when the level of plasmatic uric acid is elevated (higher than 4 mg/mL) [20]. Vitamin C can spare uric acid, which scavenges radicals and stabilizes vitamin C by iron chelation. In CLL, uric acid overproduction results from a high cell turnover rate and degradation of leukemic cells during chemotherapy. In addition, treatment of CLL with corticosteroids and allopurinol caused decreased serum levels of uric acid, sometimes under 2 mg/dL.

Progressive accumulation of leukemic lymphocytes in CLL may also be the result of decreased apoptosis and an altered age-related immune response. Oxidative stress increases immunoglobulin k light chain production in B cells and contributes to the deficiency of antibodies and hypogammaglobulinemia [9]. In our study, hypogammaglobulinemia was present in all cases in group A and in 75% of cases in group B. One third of patients had selective hypogammaglobulinemia (hypo-IgAs) which predisposes to frequent pulmonary infections. Vitamin C seems to improve the antioxidant capacity in group B where there was a 25% reduction in the number of cases associated with hypogammaglobulinemia.

Granulocytopenia was present in 60% of cases (a quarter of patients having granulocytes < 1000/mm^3^) increasing the risk of infections. Infectious complications (especially pulmonary infections) were more frequent in group A (twenty-two patients versus five patients in group B), in B or C stages of disease, who were treated with aggressive chemotherapy. Some of them were smokers (at least twenty cigarettes/day for a period of five years), or suffered from respiratory diseases, diabetes mellitus, chronic alcoholism, and severe liver disease. Infections were more frequent in the advanced stages of the disease, B and C, due to the decrease of the immune response, hypogammaglobulinemia, aggressive chemotherapy, and granulocytopenia, especially in the patients with comorbidities (diabetes mellitus, respiratory diseases, and alcoholism) and who were heavy smokers. The deficiency of the antioxidant capacity most likely modified the proliferation of lymphocytes, the functions of B cells, including antibody production and IgA mucosal immunity, increasing the risk of infections. In the B group of patients, who received immunostimulating antioxidant treatment, infections were less frequent, whereas, in the A group, which did not receive antioxidant treatment, we noted the decrease of the immune response and granulocytopenia and the alteration of the functions of lymphocytes and granulocytes all of them facilitating the development of infections. Administration of exogenous antioxidants and a healthy lifestyle (a diet with fruit, vegetables, nuts, and seeds, nonsmokers, and nondrinkers) reduced the complications due to infections in a quarter of patients with CLL from group B versus patients with CLL from group A (similar conditions of stages of CLL and therapeutic regimens) presumably due to their immunostimulating effects.

## 5. Conclusions

Oxidative stress plays a role in infections due to chronic lymphocytic leukemia. Administration of antioxidants stimulated the immune response and the tolerance to chemotherapy and decreased the number of infectious complications in CLL. A healthy lifestyle (a diet with fruit, vegetables, nuts, and seeds rich in natural antioxidants, vitamin C, vitamin E, selenium, zinc, and copper, avoiding smoking and pollutants, and limiting alcohol consumption) reduced the risk of infectious complications and may improve the quality of life for these patients.

## Figures and Tables

**Table 1:** Characteristics of the patients.

	Patients with CLL treated with antileukemic therapy (group A) Number of patients: 44	Patients with CLL treated with antileukemic therapy and antioxidants (group B) Number of patients: 40	*P*
Median age, years	47–85	51–84	NS
Male (%)	63	61	NS
Binet stage			
A	13	10	NS
B	15	15	NS
C	16	15	NS
Phenotype			
B	42	38	NS
T	2	2	NS

**Table 2:** Pro- and antioxidant status of the patients.

Pro- and antioxidant status	Patients with CLL treated with antileukemic therapy (group A) Number of patients: 44	Patients with CLL treated with antileukemic therapy and antioxidants (group B) Number of patients: 40	*P*
FORD (mmol/L)	0.48–0.96	0.39–0.81	<0.05
FORT (mmol/L)	2.7–3.8	2.5–3.2	NS
Uric acid (mg/dL)			
Normal (2–7 mg/dL)	28	16	NS
High	34	6	<0.05

**Table 3:** Antileukemic therapy and comorbidities.

Antileukemic therapy	Patients with CLL treated with antileukemic therapy (group A) Number of patients: 44	Patients with CLL treated with antileukemic therapy and antioxidants (group B) Number of patients: 40	*P*
Chl + PDN	10	10	NS
CVP	10	9	NS
Fludarabine	5	5	NS
RFC	4	4	NS
Alemtuzumab	2	2	NS
Vitamin C	—	40	NA
Smokers	19	16	NS
Comorbidities			
Arterial hypertension	3	3	NS
Respiratory diseases	5	2	<0.05
Diabetes mellitus	3	1	NS
Liver disease	2	0	NS
Kidney failure	1	0	NS
Dyslipidemic sdr.	0	2	NS
Secondary cancer	4	2	<0.05

**Table 4:** Type of infections.

Infections	Patients with CLL treated with antileukemic therapy (group A) Number of patients: 44	Patients with CLL treated with antileukemic therapy and antioxidants (group B) Number of patients: 40	*P*
	**22**	**5**	**<0.05**
Viral pneumonia	12	2	<0.05
Bacterial pneumonia	4	1	<0.05
Pleurisy	1	0	NS
Urinary infection	3	2	NS
Cutaneous infection	2	0	NS
